# High sensitivity flexible strain sensor for motion monitoring based on MWCNT@MXene and silicone rubber

**DOI:** 10.1038/s41598-025-88372-y

**Published:** 2025-01-30

**Authors:** Muhammad Luthfi Hakim, Zufar Alfarros, Herianto Herianto, Muhammad Akhsin Muflikhun

**Affiliations:** 1 Mechanical and Industrial Engineering Department, Universitas Gadjah Mada, Yogyakarta, Indonesia; 2 Department of Electrical Engineering Education, Universitas Negeri Yogyakarta, Yogyakarta, Indonesia; 3 Center for Energy Studies, Universitas Gadjah Mada, Yogyakarta, Indonesia

**Keywords:** Mechanical engineering, Biomedical engineering

## Abstract

Research on flexible strain sensors has grown rapidly and is widely applied in the fields of soft robotics, body motion detection, wearable sensors, health monitoring, and sports. In this study, MXene was successfully synthesized in powder form and combined with multi-walled carbon nanotube (MWCNT) to develop MWCNT@MXene conductive network-based flexible strain sensors with silicone rubber (SR) substrate. Combining MWCNTs with MXene as a conductive material has been shown to significantly improve the sensor performance, due to MXene’s high conductivity properties that strengthen the MWCNT conductive pathway, increase sensitivity, and improve sensor stability. The sensor is fabricated by a sandwich method consisting of three layers, which enables more accurate and reliable detection of strain changes. The main innovation of this research is the utilization of MWCNT@MXene as a conductive material that optimizes the performance of flexible strain sensors, overcomes the limitations of previous materials, and makes it a more effective solution for long-term applications. Furthermore, the sensor was evaluated to test its performance through sensitivity, linearity, response time, and durability tests. The results showed that the sensor exhibited excellent performance with a high sensitivity of 39.97 over a strain range of 0-100% and excellent linearity (0.99) over a strain of 0–50%. The sensor also has a fast response time of about 70 ms, it also has good stability during low (1–5%) and high (20–100%) strain cycle testing and can withstand up to 1200 loading and unloading cycles. In addition, the sensor effectively detects a wide range of body movements, including finger, wrist and knee movements. These findings show that the electromechanical properties of strain sensors are significantly improved through the use of MWCNT@MXene as a conductive material, so these sensors are considered a promising solution for applications in wearables and body motion monitoring.

## Introduction

In recent years, researchers around the world have become increasingly interested in wearable electronic devices for applications in the fields of electronic skin^1^, soft robotics^2^, and human motion detection^3^. Strain sensors, in particular, have been widely used to detect various body movements, including those of the fingers, neck, wrist, shoulder, and elbow^4–6^. Flexible strain sensors in this case play a role by converting strain signals into electrical signal output, so they need to have high sensitivity, fast response and recovery, and good robustness. However, strain sensors available in the market are generally rigid metal-based, limiting their flexibility and applications. To overcome this limitation, several researchers have developed flexible strain sensors. As a conductive building block, multi-walled carbon nanotubes (MWCNTs) have good electrical and mechanical properties^7–9^, so they have great potential for application in strain sensors. In addition, MXene material has outstanding electrical conductivity^10,11^, which is also promising as a key component in the development of flexible sensors. The blending of MWCNTs and MXene opens great opportunities in flexible strain sensor research as the combination of the two can produce composites with high sensitivity and good durability. By combining the mechanical strength of MWCNT and the high conductivity of MXene, the resulting sensors will be more flexible and efficient for wearable applications in various fields.

The performance of flexible strain sensors is generally evaluated based on several key parameters, namely sensitivity, linearity, response and recovery time, and durability, which are the main parameters to assess their reliability in real applications^12,13^. Studies on sensitivity, often expressed in terms of gauge factor (GF), show that nanocomposite materials such as carbon nanotubes (CNTs), graphene, graphite and carbon black have a remarkable ability to detect small changes in mechanical strain^14–17^. Like the research conducted by Sonil et al. who developed a strain sensor made from conductive graphite. The sensor shows a high sensitivity of 3247 at 50% strain and has a fast response time of 0.11 s^16^. In addition, Wang et al. also developed a flexible strain sensor based on PDA@RGO/EVA composites, the sensor also showed high sensitivity of 1801.21 and durability of 2000 cycles^18^. Linearity is also an important concern, especially to ensure that the sensor’s response to strain remains consistent and predictable. Research by Liu et al. shows that Ecoflex substrate material with carbon/graphene filler can produce a linearity of 0.9892 in the detection range of 0–90%^19^.

In addition, durability is a critical aspect that determines the ability of the sensor to withstand repeated loading and unloading cycles without losing performance. Research by Bi, et al. shows that the use of elastomeric materials such as Ecoflex that has been added with nanofillers can provide exceptional resistance to repeated cycles of stretching and releasing (6000 cycles)^20^. To date, various approaches have been applied to improve the performance of sensitivity, linearity and durability in strain sensors. However, there are still challenges in optimally integrating these three aspects to meet the needs of complex applications, especially in detecting human body movements accurately and stably. Therefore, this research seeks to explore and develop strain sensors with superior performance in all three parameters.

Flexible strain sensor substrates need to respond quickly to changes in stress elasticity, so they need substrates that are elastic, stable, and able to maintain their structural integrity despite repeated strain. Research by Zhao et al. used graphene/silicone rubber material and produced a sensor with a sensitivity of 839.02, fast response time (37 ms), and good durability^21^. In addition, Song et al. also developed a strain sensor for human body motion detection with SR/graphene/CCB material, resulting in a sensor with a sensitivity of 326 and resistance to pull-off of more than 1000 cycles^22^. Rulong et al. developed a polyacrylic acid/chitosan adhesive-based strain sensor that showed high stability up to 5.000 cycles. However, the resulting sensitivity is relatively low, at 5.7^23^. Although various materials such as graphene and chitosan have shown promising potential in the development of flexible strain sensors, the search for materials with superior performance continues. In this context, Carbon Nanotubes (CNTs) are one of the interesting candidate materials to be explored further.

MWCNT-based strain sensors have been widely developed because they have advantages such as good sensitivity, flexibility, and the ability to detect strain changes in a variety of applications, including human motion monitoring, soft robotics, and human-computer interaction^24–28^. Previous studies have shown that the nanotube-shaped structure of MWCNTs enables the formation of stable conductive pathways under strain, thereby improving the performance of the sensor^29,30^. The use of conductive fillers such as CNTs and Mxene in strain sensor materials has been investigated by Yu et al. The resulting sensor showed good performance, with sensitivity reaching 5.59, response time of 330 ms, and durability of up to 100 test cycles^10^. However, although the sensitivity of MWCNT and Mxene-based sensors is excellent, there are still limitations in terms of long-term stability and sensitivity over a wider strain range. To overcome these limitations, this study proposes a new approach with the incorporation of Mxene in MWCNT-based composites using a sandwich method and an innovative mixing technique. The sandwich method allows for a more homogeneous distribution of MWCNTs and Mxene, creating a more stable conductive pathway, while the mixing of the materials synergistically enhances the interaction between the conductive fillers. This combination not only increases the sensitivity of the sensor but also improves the stability of the sensor under repeated strain cycles, making it more reliable for long-term applications. The innovative contribution of this research lies in the application of sandwich-based fabrication techniques and Mxene-MWCNT blending, which have not been widely applied in previous literature, as a novel solution to maximize the sensitivity and durability of the sensor over a wider strain range.

This research introduces a simple and efficient method for designing flexible strain sensors that can be applied in human body motion monitoring. MWCNT and MXene materials are used as conductive components due to their outstanding conductivity, while silicone rubber is chosen as the substrate due to its superior mechanical properties in maintaining structural integrity. The sensor was then evaluated through electromechanical properties testing to assess its performance.

## Materials and method

### Materials and characterization

 The materials used in this study include MXene obtained from NRE Lab, Medan, Indonesia. MWCNTs with a diameter of 3–5 nm and a length of 8–15 nm produced in Mainland, China. Silicone Rubber (SR) purchased from PT Grasindo Multi Sentosa, Indonesia, and isopropanol (IPA) obtained from Sigma-Aldrich, USA. Material characterization was carried out using several equipment. Measurement of electrical properties was carried out with the LCR meter UNI-T UT61E+ (China). Chemical structure analysis was performed using Fourier-transform infrared spectroscopy (FTIR) IRXross from Shimadzu. Elemental mapping and morphological observations of the materials were performed using a Scanning Electron Microscope-Energy Dispersive X-ray Spectroscopy (SEM-EDS) Phenom Pro X6 from Thermo Scientific.

### Synthesis Mxene

**Fig. 1:**
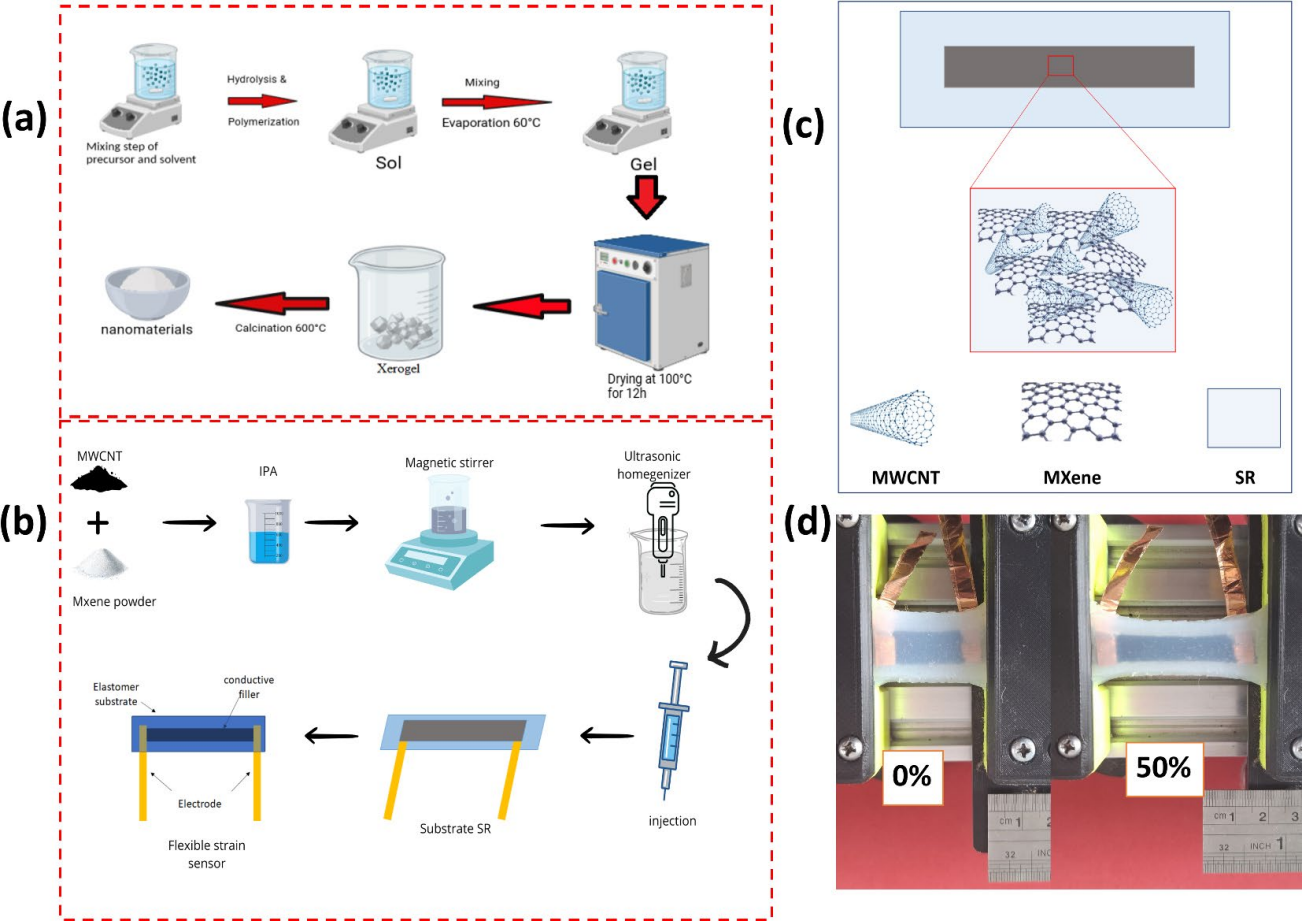
(**a**) Illustration of MXene powder manufacturing process (**b**) MWCNT@MXene sensor manufacturing (**c**) illustration of MWCNT@MXene material mixture and (**d**) initial position and 50% strain.

 The synthesis of MXene nanomaterials using the sol-gel method is based on the approach reported in a previous study by Utomo et al.^31^. This method involves controlled stages of chemical reactions to produce materials with homogeneous nanostructures and optimal functional properties. The complete stages of this synthesis process can be seen in Fig. 1a. First, prepare Solution A by mixing 4 ml of tetraethyl orthosilicate (TEOS) as a precursor with 1 ml of ammonia and 30 ml of ethanol, then stirring for 5 min. Next, prepare the hydrolysis catalyst by mixing 3 ml HNO3 and 150 ml H2O, then stirring for 5 min. Combine Solution A and the hydrolysis catalyst and stir for 120 min at 60 °C, resulting in a sol. Solution B was prepared by mixing 4 ml of Mxene as the next precursor. The TEOS and Mxene solutions were then mixed and stirred for 120 min at 60 °C to transform the material into a gel phase. Once formed, the gelled material was dried to remove the solvent, with a drying temperature of 100 °C for 12 h. It was then calcined for 3 h at 600 °C to improve crystallinity. The powder formed was pulverized by the pounding process and filtered using 200 and 600 mesh size sieves, resulting in high-quality Mxane nanomaterials and uniform particle distribution.

### Preparation of MWCNT@Mxene

 The manufacture of flexible strain sensors involves the design and manufacture of composite materials that are capable of detecting strain changes with high accuracy. The composite material used is generally a combination of conductive material as filler and flexible material as elastomer. In this research, Silicone rubber (SR) was chosen as the elastomer because it has high flexibility and durability. As a conductive filler material, MWCNT and Mxane were chosen because they have high sensitivity and conductivity. The manufacturing step of the flexible strain sensor begins with mixing 0.1 g of Mxane powder and 0.4 g of MWCNT, then dissolved using 20 ml of isopropyl alcohol (IPA) liquid. The liquid mixture was stirred using a magnetic stirrer for 10 min at 500 rpm. After stirring, the mixture was dispersed using an ultrasonic homogenizer with a power of 300 W for 10 min, which aims to ensure that the conductive liquid is evenly dispersed. After that, the conductive carvings were put into a pipette as much as 0.7 ml for the next stage of preparation. Illustration of the preparation of MWCNT@Mxane conductive liquid can be seen in Fig. 1b.

### Preparation of SR/MWCNT@Mxene strain sensors

 The flexible strain sensor mold is made of acrylic that has been processed using laser cutting with dimensions of 40 × 20 × 5 mm. Next, the flexible strain sensor was fabricated using a sandwich method consisting of three layers. The first layer, which is the base layer, consists of silicone rubber that serves as a flexible and elastic substrate. On top of the silicone rubber layer, copper-based electrodes are attached with adhesive, which serves as an electrically conductive path. Next, the middle layer is filled with 0.7 ml of MWCNT@MXene composite material-based conductive ink that has been processed as shown in Fig. 1b. The mixture of MWCNT and MXene in this ink is designed to form a conductive network that is stable and efficient in conducting electricity under strain. As a protective and sealing agent, the last layer consists of silicone rubber, which serves to cover the conductive ink layer while protecting the entire sensor structure from external damage. A visual illustration of the MWCNT and MXene material mixture in the conductive ink can be seen in Fig. 1c.

### Experimental setup

 The performance testing process of the flexible strain sensor was conducted using an electromechanical testing machine developed by Hakim et al.^32^, which is designed to provide precise control in strain application. The testing involved evaluating several key performance parameters, namely sensitivity, linearity, response time and recovery time, and sensor durability. Sensitivity measures the ability of the sensor to detect changes in strain through changes in the electrical signal generated, while linearity evaluates the consistency of the relationship between the applied strain and the sensor response. Response time and recovery time were tested to determine the speed at which the sensor responds to strain and returns to its initial state after the strain is released, which is critical for dynamic applications. In addition, the durability of the sensor is tested through repeated strain cycles to ensure long-term performance stability. During the test, the sensor was placed on a testing machine with controlled strain, and an overview of the sensor in its initial state (no strain) as well as at 50% strain can be seen in Fig. 1d, providing a visualization of the change in shape of the sensor during the test.

## Results

### SEM and FTIR

**Fig. 2:**
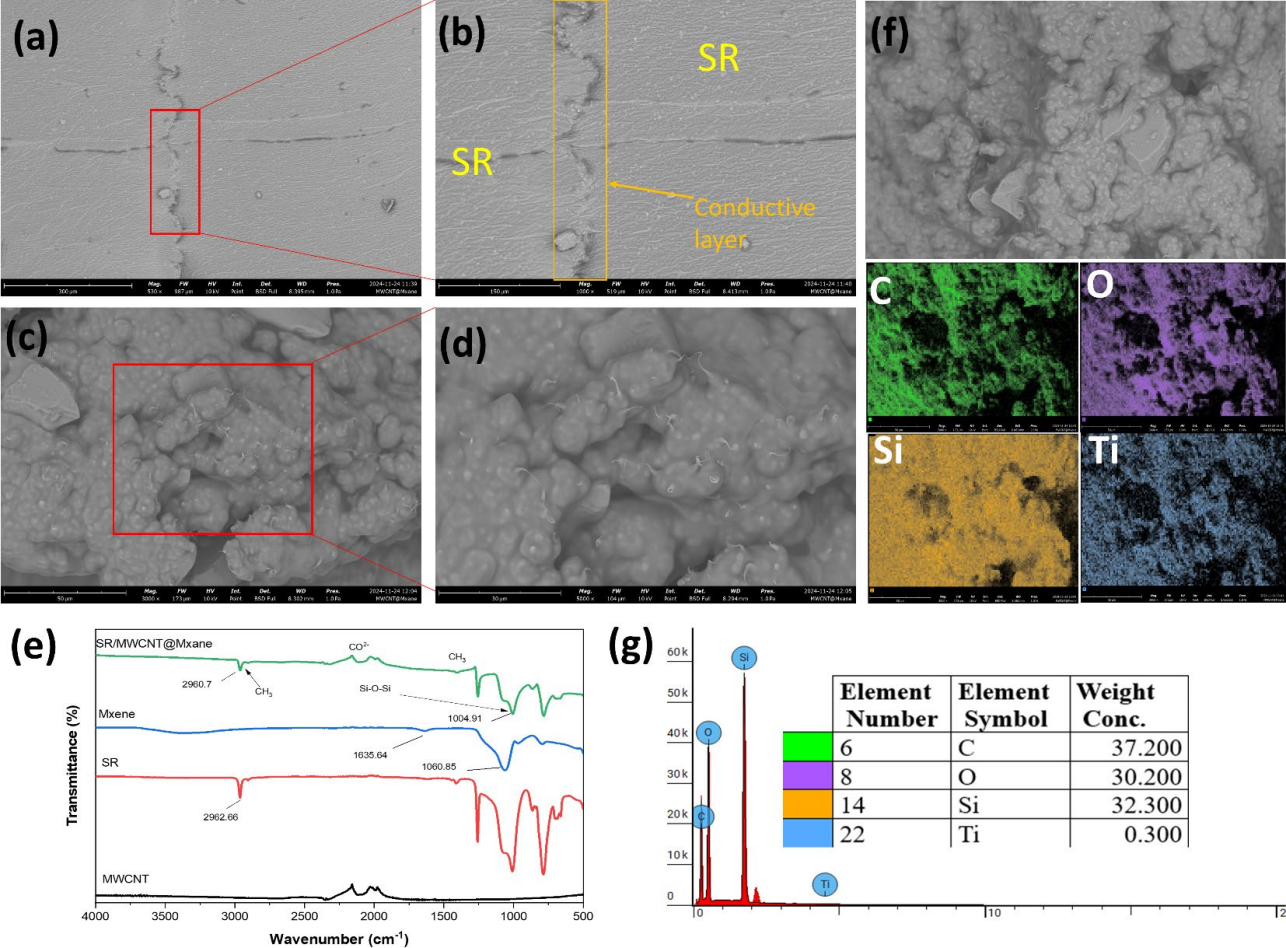
(**a**) 3-layer sensor, (**b**) 1000x magnification, (**c**) MWCNT@Mxene conductive network, (**d**) 5000x magnification, (**e**) FTIR, (**f**) EDS mapping and (**g**) elemental distribution.

 The SEM images of the MWCNT@MXene-based flexible strain sensor with an SR substrate, as illustrated in Fig. 2a,b, reveal a carefully constructed three-layer architecture. The top and bottom layers are made of silicone rubber (SR), chosen for its mechanical flexibility and stability, while the middle layer comprises the MWCNT@MXene conductive network. This sandwich-like structure is designed to balance mechanical robustness and electrical performance, making it ideal for applications in wearable strain sensors. Figure 2c,d show magnified images of the MWCNT@Mxene conductive layer. In Fig. 2c the MWCNTs are perfectly dispersed. This can improve the electromechanical properties of the strain sensor, as the even distribution of MWCNTs creates a more consistent conductive path in the silicon matrix. Good dispersion also minimizes the formation of agglomerations, which can reduce the sensitivity and stability of the sensor during use. Thus, the sensor can exhibit a better linear response to strain and has higher sensitivity^7,24^. Figure 2e presents the FTIR analysis results of MWCNT, SR, MXene, and SR/MWCNT@MXene composites. After the incorporation of SR with MWCNT/MXene, the resulting composite does not show the formation of new chemical bands, and the existing bands remain consistent with the original components. However, the intensity of the characteristic peaks at around ∼2962 and ∼2904 cm^−1^ decreased. In addition, the intensity of the peak at ∼2085 cm^−1^, which indicates CO_2−_ bonds, also decreased. The peak at ∼1257 cm^−1^ was identified as symmetric bending of the CH_2_ bond, while the peaks at ∼1066 cm^−1^ and ∼1006 cm^−1^ were attributed to symmetric and asymmetric stretching of the Si-O-Si bond, respectively^33^. EDS mapping (Fig. 2f) identified four main elements: C, O, Si, and Ti, with an even distribution of C and Ti elements in the MWCNT@MXene conductive layer. This indicates a good distribution of the filler, which contributes to the improved sensitivity of the sensor. The distribution of these elements is shown in more detail through the EDS spectra in Fig. 2g.

### Strain-sensing characteristic

The performance of strain sensors MWCNT@Mxane-0.7 in detecting strain as well as their electromechanical response under tensile strain and durability test conditions have been discussed in detail. The initial resistance value of the sensor, denoted as R0, is one of the key parameters for assessing sensing performance. The change in resistance is expressed as ΔR, which is calculated from the difference of R-R0​. Meanwhile, the strain value is denoted by ε. Thus, the sensitivity gauge factor (GF) is formulated as Eq. 1^34^. The equation describes the sensitivity of the sensor to the applied strain.

1 $$\:\text{G}\text{F}=\:\frac{{\Delta\:}\text{R}/\text{R}0\text{}}{{\upepsilon\:}}$$

**Fig. 3:**
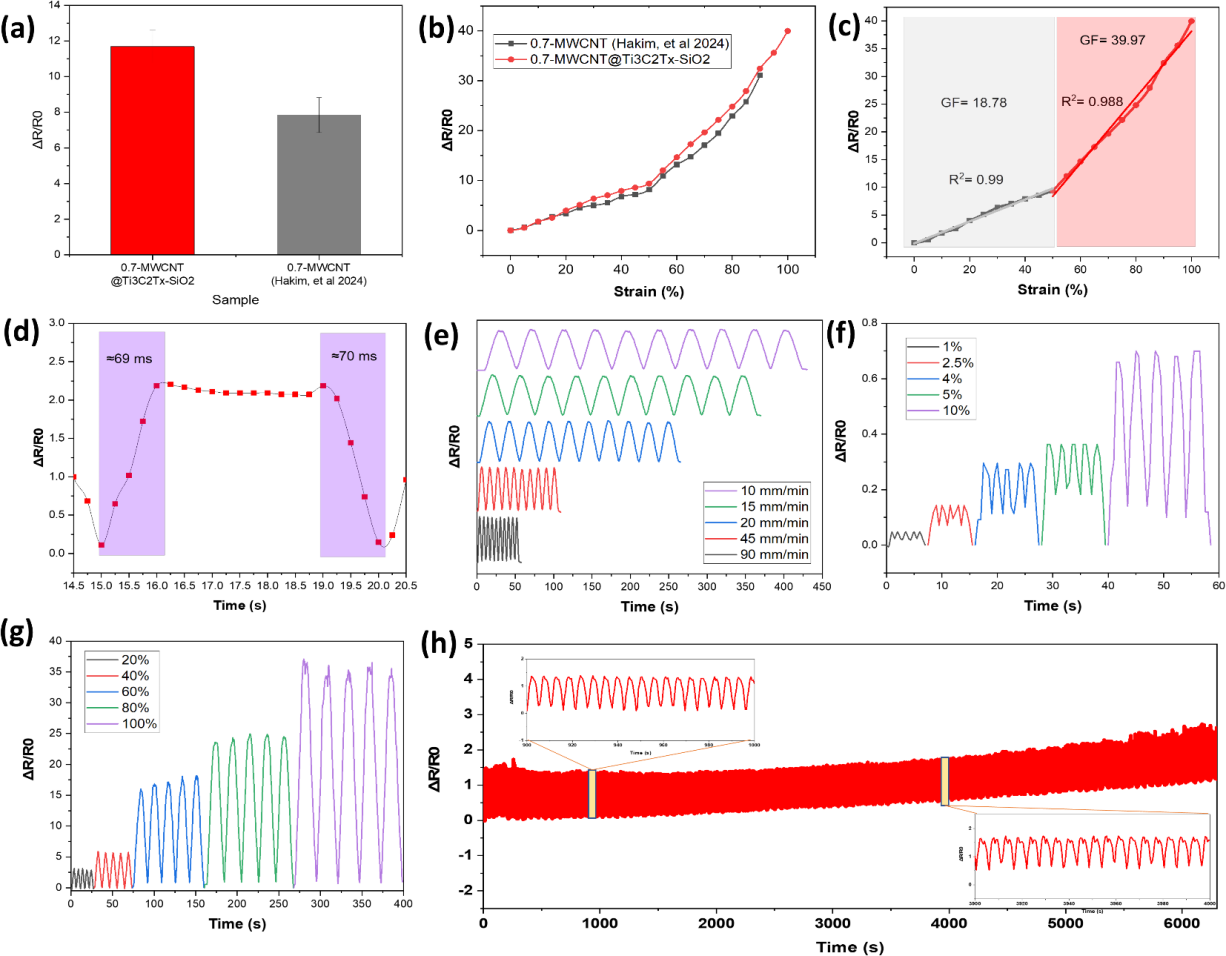
(**a**) Resistance values of MWCNT@MXene sensor compared to MWCNT-0.7^31^, (**b**) Relative resistance values of MWCNT@MXene and MWCNT-0.7^31^ strain sensors at different strains. (**c**) sensitivity and linearity values (**d**) response and recovery time values. (**e**) Relative resistance values at different speed variations. (**f**) Sensor performance at small strains (1–10%) (**g**) large strain (20–100%) and (**h**) Durability.

The resistance value of the MWCNT@Mxene-0.7 strain sensor shows an improvement compared to the research of Hakim et al. on MWCNT-0.7^32^, with the resistance value reaching about 11.8 kΩ (Fig. 3a). In addition, the sensitivity value of the MWCNT@Mxene-0.7 sensor is also higher than that of MWCNT-0.7, indicating that the MWCNT@Mxene-0.7 composite is more responsive to strain changes (Fig. 3b). The figure shows that the relative resistance value (ΔR/R) increases as the strain on the sensor increases. This occurs due to crack propagation caused by the increase in strain. As a result, the resistance increases significantly, which results in a sharp increase in sensitivity^5^. The developed sensor showed excellent sensitivity, with a value of 39.97 and linearity of 0.99 over a strain range of 0–50% (Fig. 3c). Such sesnitivity values are superior to previously reported flexible strain sensors (12.1 and 26.7)^35,36^. It also has fast response and recovery times of about 69 ms and 70 ms, respectively (Fig. 3d), indicating that it can quickly detect strain changes in dynamic applications.

In Fig. 3e the strain sensor MWCNT@Mxene-0.7 also shows stable relative resistance values in the tensile and release speed tests at variations of 10, 15, 20, 45 and 90 mm/min. This indicates that the sensor has good resistance to changes in speed in repeated applications. The sensor can also detect resistance changes at small strains, such as 1%, 2.5%, 4%, 5 and 10% (Fig. 3f), indicating sensitive detection capability at low strains. In addition, the sensor remains capable of detecting resistance changes at high strains, such as 20%, 40%, 60%, 80%, and 100% (Fig. 3g), indicating a wide detection range. In addition, the sensor MWCNT@Mxene-0.7 showed consistent performance after 1,200 loading and unloading cycles at a strain of 20%, indicating good stability and resistance to mechanical cycling (Fig. 3h). The figure shows an increase in relative resistance starting at a time of 4,000 s, or about 800 loading and unloading cycles. This could be due to shifting of the conductive material, deformation of the MWCNT@Mxene nanostructure, weakening of the adhesion between materials, formation of micro-cracks, or relaxation of the elastomeric material due to repeated loading and unloading cycles, resulting in an increase in the relative resistance of the sensor^37–39^. This indicates that the sensor is reliable for long-term applications without significant performance degradation. With this analysis, it can be concluded that the addition of Mxene significantly improves the performance of the material, especially in terms of sensitivity, stability and durability. These results are also consistent with findings from previous studies^40^.

Table 1 presents a comparison of sensor performance against previous research. The sensors listed in the table use various fabrication methods and materials, which have an impact on their performance. For example, the sensor developed by Yang et al. with conductive RGO/Ag nanoparticles yielded a high sensitivity value of 475, but was limited to a strain range of 14.5%^41^. In addition, graphene conductive material was used by Tian et al. to make strain sensors. The sensor made has a lower sensitivity value of 9.49 in the detection range of 10%^42^. In contrast, Hakim et al. developed a strain sensor with conductive material in the form of MWCNT which has a faster response time, which is about 65 ms. however, the resulting sensitivity reached 34.47 at a detection range of 90%^32^. The sensor developed by Wang et al. also has good stability resistance at 2500 cycles (loading and unloading), but the sensor still has the disadvantage that the response time is relatively long, which is around 280 ms^43^. The direct writing printing method was used by Chen et al. to make strain sensors, the material used was graphene/carbon nanotube aerogel. The resulting sensor has a sensitivity of 18.55 in the detection range of 20%^44^. In general, each sensor developed has its own advantages in terms of performance. In our study, the development of MWCNT@Mxene-based sensors showed high sensitivity of 39.97 at 100% detection range, as well as good stability up to 1,200 cycles. The addition of Mxene contributes to the improved sensitivity and stability of the sensor due to its high conductivity properties and its ability to form a more efficient conductive network. Recent research on flexible strain sensors has shown that they have good durability, being able to withstand up to 5,000 loading and unloading cycles. However, they have the disadvantage of a relatively slow response time, which ranges from 110 to 300 ms^16,19,23^.

**Table 1:** Comparison of MWCNT@Mxane-0.7 sensors with previous research.

No	Materials	GF (strain range (%))	Linearity	Respons (ms)	Durability (cycle)	Ref
1	RGO/Ag nanoparticles	475 (14.5)	-	-	500	^41^
2	Graphene	9.49 (10)	-	-	150	^42^
3	MWCNT	34.47 (90)	0.99	65	1200	^32^
4	CNT	12.88 (160)	-	280	2500	^43^
5	Graphene/Carbon nanotube aerogel	18.55 (20)	-	-	900	^44^
6	Graphite pencils	3247 (50)	-	110	5000	^16^
7	Carbon/graphene	35(100)	0.9892	200	5000	^19^
8	Conductive hydrogels	5.7(500)	0.99	300	5000	^23^
9	Nano-Ag/graphene	492.95(16)	-	-	1000	^15^
10	3D MXene and 1D CNTs	5.59 (110)	-	330	100	^10^
11	MWCNT@Mxene-0.7	39.97 (100)	0.99	70	1200	This work

### Human motion detection

**Fig. 4:**
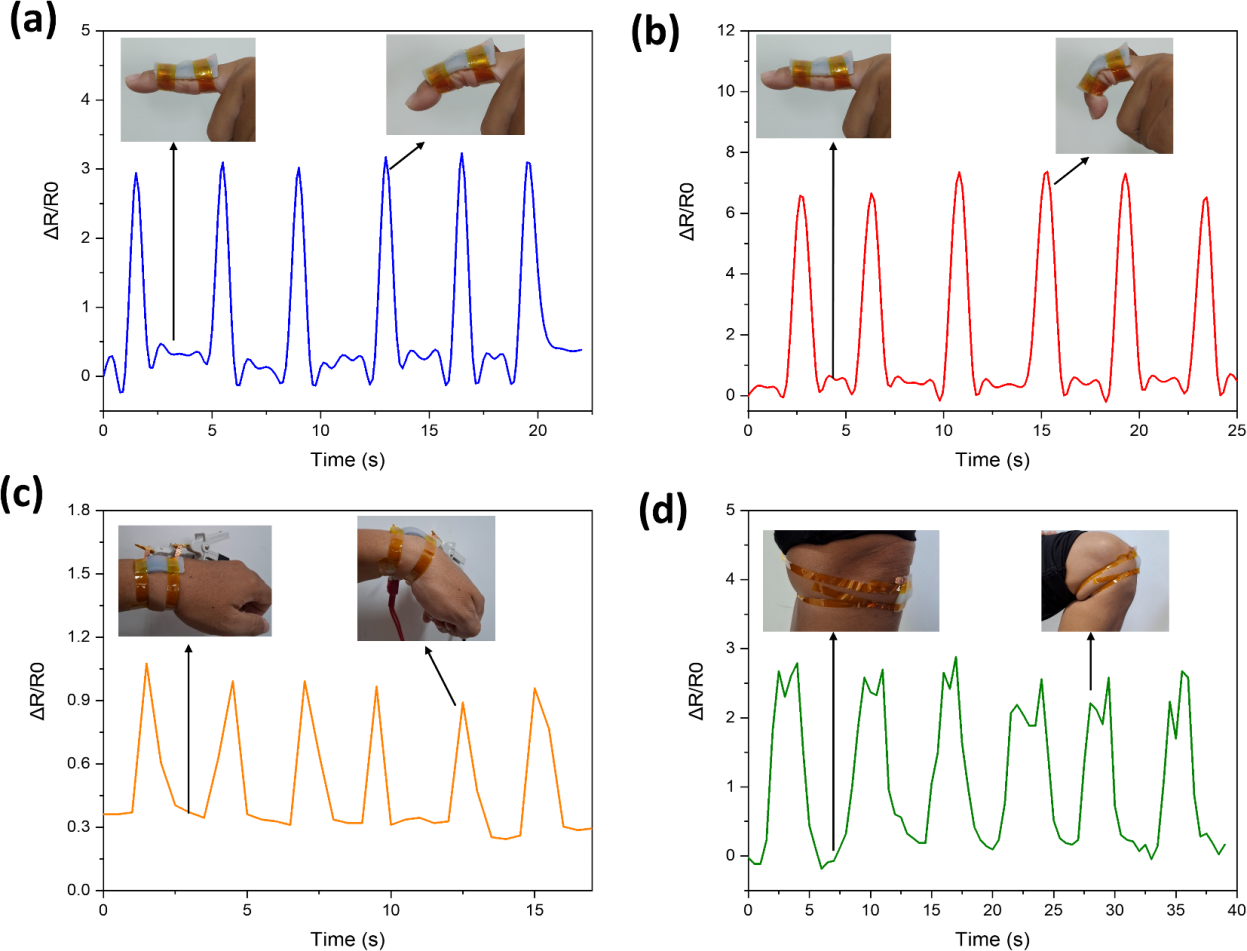
Application of the sensor on (**a**) medium bend finger, (**b**) full bend finger, (**c**) wrist and (**d**) knee.

 Flexible strain sensors MWCNT@Mxene-0.7 deliver high sensitivity and linearity, fast response time, and stable loading and unloading resistance, making them suitable for applications to detect various body movements. The sensor works by converting body movements into electrical signals, enabling accurate interpretation of movements for various applications. Figure 4 shows the application of the MWCNT@Mxene-0.7 sensor to detect body movements such as fingers, wrists and knees. From the figure, the sensor quickly captures any defromation caused by movement and then converts it into electrical signals. To effectively assess hand function to monitor human health, we explored the potential use of strain sensors applied to the fingers (Fig. 4a,b). Figure 4a shows the performance of the strain sensor in detecting bending of the finger at a moderate bending position. While Fig. 4b shows full finger flexion. The relative resistance values show good stability when the sensor is released and bent. In addition, we evaluated the ability of the sensor to detect the wrist as shown in Fig. 4c. It was observed that the sensor response behavior was stable when repeated for 6 cycles. We installed the sensor for a significant movement, which is to detect the movement of the knee when it is bent (Fig. 4d). From the figure, it can be seen that the sensor response has the same pattern when the knee is bent and then released 6 times. This shows that the sensor MWCNT@Mxene-0.7 has a remarkable ability to detect various movements of human body parts.

## Conclusions

We have successfully developed a flexible strain sensor based on MWCNT@MXene-0.7 conductive network with SR substrate. The sensor exhibits a high sensitivity of 39.97 over the 0-100% strain range, with excellent linearity (0.99) over 0–50% strain. In addition, the sensor has a fast response time of about 70 ms, stable performance during cycle repetition at both low (1–5%) and high (20–100%) strains and can withstand up to 1200 loading and unloading cycles. The sensor is also effectively used to detect various body movements, such as finger, wrist, and knee movements. The results of this study prove that the addition of MXene conductive material significantly improves the electromechanical performance of the strain sensor. We have also made observations using Scanning Electron Microscopy (SEM), which shows that the distribution of MWCNT and MXene networks in the SR matrix is very homogeneous. The layered structure of MXene is seen to form an efficient conductive pathway, while MWCNTs create a nano-network connection that strengthens the mechanical stability and improves the sensitivity of the sensor. The results of this study prove that the addition of MXene conductive material significantly improves the electromechanical performance of strain sensors.

## Data Availability

The data are available upon request to the corresponding author.

## References

[1] Zhou, J. et al. Highly sensitive and stretchable strain sensors based on serpentine-shaped composite films for flexible electronic skin applications. *Compos. Sci. Technol.* **197**, 108215 (2020).

[2] Xu, J. et al. Buckling-inspired triboelectric sensor for multifunctional sensing of soft robotics and wearable devices. *Nano Energy* **130**, 110141 (2024).

[3] Mahato, R., Masiul Islam, S. & Singh, S. Flexible piezo-resistive strain sensors based on silver nanowires and graphene nanoplatelets reinforced polydimethylsiloxane for human motion detection. *Mater. Today Commun.* **40**, 110056 (2024).

[4] Tan, C. et al. A high performance wearable strain sensor with advanced thermal management for motion monitoring. *Nat. Commun.* **11**, 3530 (2020). 32669576 10.1038/s41467-020-17301-6 PMC7363829

[5] Sun, H. et al. An ultrasensitive and stretchable strain sensor based on a microcrack structure for motion monitoring. *Microsystems Nanoeng.* **8**, 111 (2022). 10.1038/s41378-022-00419-6 PMC9522852 36187892

[6] Li, Y. et al. Hybrid strategy of graphene/carbon nanotube hierarchical networks for highly sensitive, flexible wearable strain sensors. *Sci. Rep.* **11**, 21006 (2021). 34697336 10.1038/s41598-021-00307-5 PMC8546066

[7] Yu, T., Lü, X. & Bao, W. High electrical self-healing flexible strain sensor based on MWCNT- polydimethylsiloxane elastomer with high gauge factor and wide measurement range. *Compos. Sci. Technol.* **238**, 110049 (2023).

[8] Zhou, Z. et al. Flexible and self-adhesive strain sensor based on GNSs/MWCNTs coated stretchable fabric for gesture monitoring and recognition. *Sens. Actuators Phys.* **349**, 114004 (2023).

[9] Haseeb ur Rehman, M., Nazar, R., Yasin, S., Ramzan, N. & Habib, M. S. Development of PANI-TPU/MWCNTs based nanocomposites for piezoresistive strain sensing applications. *Mater. Lett.* **328**, 133110 (2022).

[10] Yu, T. et al. Development of a flexible strain sensor for monitoring human activities based on 3D MXene microspheres and 1D CNTs. *Mater. Lett.* **377**, 137460 (2024).

[11] Fu, W. et al. Highly stretchable and conductiveMXene/polyurethane composite film coated on various flexible substrates for ultrasensitive strain sensors. *Mater. Lett.* **320**, 132328 (2022).

[12] Yi, H., Wang, S., Mei, S. & Li, Z. Conductive polymer composites for resistive flexible strain sensors. *Polym. (Guildf)* **307**, 127286 (2024).

[13] Khalid, M. A. U. & Chang, S. H. Flexible strain sensors for wearable applications fabricated using novel functional nanocomposites: A review. *Compos. Struct.* **284**, 115214 (2022).

[14] Liu, J. et al. Engineering PDA@CNTs-enhanced sulfobetaine methacrylate hydrogels for superior flexible sensor applications. *Polym. (Guildf)* **311**, 127482 (2024).

[15] Tseng, S. F., Tsai, H. T., Lee, C. C. & Kuo, C. C. Laser-induced nano-Ag/graphene composites for highly responsive flexible strain sensors. *Compos. Part. Appl. Sci. Manuf.* **188**, 108586 (2025).

[16] Sonil, N. I., Ullah, Z., Chen, J. & Wang, G. P. Wearable strain sensors for human motion detection and health monitoring based on hybrid graphite-textile flexible electrodes. *J. Mater. Res. Technol.* **26**, 764–774 (2023).

[17] Huang, J. et al. Fabrication of styrene–butadienestyrene (SBS) matrix-based flexible strain sensors with brittle cellulose nanocrystal (CNC)/carbon black (CB) segregated networks. *Compos. Struct.* **320**, 117231 (2023).

[18] Wang, S. et al. Ultra-fast light repair, ultrasensitive, large strain detection range PDA@RGO/EVA composites fiber flexible strain sensor. *Sens. Actuators Phys.* **377**, 115755 (2024).

[19] Lu, Z. et al. High-performance multidirectional flexible strain sensor for human motion and health monitoring. *ACS Appl. Mater. Interfaces* **16**, 41409–41420 (2024). 39074313 10.1021/acsami.4c04583

[20] Bi, Z., Sun, Q., Tang, C., Wu, H. & Lu, Y. Highly sensitive, repairable, and flexible strain sensors with a wide sensing range based on an EG/Sn–Bi/EG-encapsulated sandwich structure. *ACS Appl. Electron. Mater.* **6**, 6698–6707 (2024).

[21] Zhao, Y., Yang, Y., Wan, B., Ding, T. & Sha, X. Improvement of shoulder peak effect in graphene/silicone rubber strain sensors by nanosilica. *Case Stud. Constr. Mater.* **21**, e03551 (2024).

[22] Song, P., Wang, G. & Zhang, Y. Preparation and performance of graphene/carbon black silicone rubber composites used for highly sensitive and flexible strain sensors. *Sens. Actuators Phys.* **323**, 112659 (2021).

[23] Lv, R. et al. A highly stretchable, self-healing, self-adhesive polyacrylic acid/chitosan multifunctional composite hydrogel for flexible strain sensors. *Carbohydr. Polym.* **123111**10.1016/j.carbpol.2024.123111 (2024). 10.1016/j.carbpol.2024.123111 39779019

[24] Bharadwaj, S. et al. Long length MWCNT/TPU composite materials for stretchable and wearable strain sensors. *Sens. Actuators Phys.* **357**, 114364 (2023).

[25] Luo, X. et al. Multi-walled carbon nanotube-enhanced polyurethane composite materials and the application in high-performance 3D printed flexible strain sensors. *Compos. Sci. Technol.* **257**, 110818 (2024).

[26] Yang, Y. et al. MWCNTs / PDMS composite enabled printed flexible omnidirectional strain sensors for wearable electronics. *Compos. Sci. Technol.* **226**, 109518 (2022).

[27] Jiang, C. S., Lv, R. Y., Zou, Y. L. & Peng, H. L. Flexible pressure sensor with wide pressure range based on 3D microporous PDMS/MWCNTs for human motion detection. *Microelectron. Eng.* **283**, 112105 (2024).

[28] Zhang, D. et al. Flexible piezoresistive sensor constructing from ILs/MWCNTs/PVDF ternary composite for high sensitivity and wide detection range. *Sens. Actuators Phys.* **367**, 115037 (2024).

[29] Feng, C. et al. Nanocellulose and multi-walled carbon nanotubes reinforced polyacrylamide/sodium alginate conductive hydrogel as flexible sensor. *J. Colloid Interface Sci.* **677**, 692–703 (2025). 39159524 10.1016/j.jcis.2024.08.067

[30] Zhang, X. et al. A high sensing performance piezoresistive sensor based on TPU/c-MWCNTs/ V2CTX-MXene electrospun film for human motion detection. *J. Alloys Compd.* **1007**, 176408 (2024).

[31] Utomo, R. S. B., Sentanuhady, J. & Muflikhun, M. A. TiO2–SiO2 nanocomposite via a novel sol-gel method with the addition of an energy monitoring device: Synthesized, characterization and anti-bacterial applications. *Ceram. Int.* **50**, 23367–23378 (2024).

[32] Hakim, M. L., Muflikhun, M. A. & Herianto & Next-gen strain sensors: Self-healing, ultra-sensitive, lightweight, and durable MWCNT-silicone rubber for advanced human motion tracking. *J. Eng. Res.*10.1016/j.jer.2024.10.009 (2024).

[33] Chen, C., Bu, X., Feng, Q. & Li, D. Cellulose nanofiber/carbon nanotube conductive nano-network as a reinforcement template for polydimethylsiloxane nanocomposite. *Polymers* **10** . 10.3390/polym10091000 (2018). 10.3390/polym10091000 PMC6403898 30960925

[34] Zhang, X. et al. High-performance flexible strain sensors based on biaxially stretched conductive polymer composites with carbon nanotubes immobilized on reduced graphene oxide. *Compos. Part. Appl. Sci. Manuf.* **151**, 106665 (2021).

[35] Abedheydari, F., Sadeghzadeh, S., Saadatbakhsh, M., Heydariyan, A. & Khakpour, E. Silver-decorated laser-induced graphene for a linear, sensitive, and almost hysteresis-free piezoresistive strain sensor. *Sci. Rep.* **14**, 28715 (2024). 39567615 10.1038/s41598-024-80158-y PMC11579415

[36] Iqra, M., Anwar, F., Jan, R. & Mohammad, M. A. A flexible piezoresistive strain sensor based on laser scribed graphene oxide on polydimethylsiloxane. *Sci. Rep.* **12**, 4882 (2022). 35318353 10.1038/s41598-022-08801-0 PMC8941115

[37] Kouediatouka, A. N. et al. Highly sensitive and durable crack structures on flexible, friction-resistant substrates. *Appl. Surf. Sci.* **685**, 161826 (2025).

[38] Xiang, D. et al. Preparation and performance of biaxially stretched CNT-SBS/POE flexible strain sensor with a double percolation structure. *Sens. Actuators Phys.* **371**, 115340 (2024).

[39] Li, L. et al. One-dimensional hierarchically structured strain sensor with high sensitivity, stretchability and durability for physiological monitoring. *Mater. Res. Bull.* **177**, 112876 (2024).

[40] Yang, C. et al. Stretchable MXene/carbon nanotube bilayer strain sensors with tunable sensitivity and working ranges. *ACS Appl. Mater. Interfaces* **16**, 30274–30283 (2024). 38822785 10.1021/acsami.4c04770

[41] Yang, Z. et al. Simultaneously detecting subtle and intensive human motions based on a silver nanoparticles bridged graphene strain sensor. *ACS Appl. Mater. Interfaces* **10**, 3948–3954 (2018). 29281246 10.1021/acsami.7b16284

[42] Tian, H. et al. Scalable fabrication of high-performance and flexible graphene strain sensors. *Nanoscale* **6**, 699–705 (2014). 24281713 10.1039/c3nr04521h

[43] Wang, G. et al. Thermoplastic polyurethane/carbon nanotube composites for stretchable flexible pressure sensors. *ACS Appl. Nano Mater.* **6**, 9865–9873 (2023).

[44] Chen, C. et al. Fabricating flexible strain sensor with direct writing graphene/carbon nanotube aeroge. *ACS Appl. Electron. Mater.*10.1021/acsaelm.2c01338 (2023).36873261

